# Double-Staged Sleeve Resection of Laryngotracheal Tumor of Papillary and Follicular Thyroid Carcinoma

**DOI:** 10.7759/cureus.25999

**Published:** 2022-06-16

**Authors:** Rachel Lim, Ikram Hakim, Mawaddah Azman, Nani Md Latar, Rohaizak Muhammad, Marina Mat Baki

**Affiliations:** 1 Otolaryngology - Head and Neck Surgery, Universiti Kebangsaan Malaysia Medical Centre, Kuala Lumpur, MYS; 2 Surgery, Universiti Kebangsaan Malaysia Medical Centre, Kuala Lumpur, MYS

**Keywords:** laryngotracheal infiltration, sleeve resection, tracheal resection, thyroid carcinoma, laryngotracheal tumor

## Abstract

Advanced thyroid carcinoma involving the upper aerodigestive tract confers a poor prognosis mainly due to airway complications. The management of thyroid carcinoma with infiltration to the aerodigestive tract has been widely discussed with no consensus regarding the best surgical technique. Complete surgical resection is the aim of the surgery. However, it has high morbidity if the postsurgical care is compromised, which will lead to airway obstruction, bleeding, infection, and anastomotic dehiscence. In our center, complete resection was achieved through cricotracheal window resection with partial closure and tracheostomy tube insertion. This procedure was chosen due to the time-sensitive nature of surgery in these patients with airway compromise and postoperative limitation of intensive care unit (ICU) bed availability. In our case series, we present six cases of papillary and follicular thyroid carcinoma complicated with intraluminal laryngotracheal infiltration and discuss its management and outcome.

## Introduction

Thyroid cancer has been increasingly prevalent in the recent decade. Poor prognostic factors in thyroid cancer include age more than 55 years, male sex, extrathyroidal extension, distant metastasis, and lymphatic invasion [1,2]. Infiltration of thyroid cancer to the surrounding structures is associated with increased rates of locoregional recurrence, distant metastasis, and higher mortality [3]. Surgical management of thyroid carcinoma infiltrating into the aerodigestive tract has been widely debated [4]. Here, we present five cases of papillary thyroid carcinoma (PTC) and one case of follicular thyroid carcinoma (FTC) with subglottic and tracheal infiltration undergoing laryngotracheal sleeve resection, partial anastomosis, and tracheostomy after total thyroidectomy, a double-staged approach. The surgical management and outcomes are discussed.

## Materials and methods

A retrospective single-center study was conducted reviewing patients diagnosed with thyroid carcinoma complicated with intraluminal laryngotracheal infiltration from 2016 to 2020 in a tertiary institution. Patients who were inoperable for curative intention or diagnosed with double synchronous tumor were excluded from the review. During this period, eight patients were treated for thyroid carcinoma with airway infiltration. One patient was excluded due to advanced local disease which could not be fully resected while another patient was excluded for having a double synchronous tumor. Out of the six patients included in the study, five had PTC while one patient was diagnosed with FTC. The medical records were reviewed, including the presentation of the patient, the extent of laryngotracheal intraluminal infiltration on endoscopy, radiological findings, staging, and surgery. Informed consent was obtained for all patients.

All patients underwent contrast-enhanced computed tomography (CT) scan of the neck and upper airway endoscopy under local anesthesia using 10% lidocaine spray to evaluate the extent of intraluminal tumor infiltration. The findings were essential for the planning of airway management perioperatively. Based on the preoperative airway endoscopy, three patients had significant intraluminal tumor invasion which required debulking before proceeding with transoral endotracheal intubation. These three patients underwent direct laryngoscopy with tubeless jet ventilation, debulking of the tumor using a laryngeal microdebrider, followed by transoral endotracheal intubation. The remaining three patients were intubated by the anesthetist with a smaller endotracheal tube without any prior debulking of the intraluminal tumor. We proceeded with total thyroidectomy and sleeve resection of the trachea with partial anastomosis and tracheostomy for all patients.

## Results

Patient characteristics

The patients in this series were aged between 44 and 74 years upon presentation. Three patients (50%) were female and the remaining three (50%) were male. Five patients were newly diagnosed with PTC, except for the sixth patient who was diagnosed with FTC. The clinical data are summarized in Table 1.

**Table 1:** Clinical data for six patients with thyroid carcinoma with airway infiltration M: male; F: female; PTC: papillary thyroid carcinoma; FTC: follicular thyroid carcinoma

No	Age/Gender	Presentation (months)	Stridor	Staging	Histology	Endoscopic airway	Initial airway	Direct laryngoscopy and intraluminal tumor debulking	Endotracheal tube size	Neck dissection	Additional procedures
1	64/M	Hemoptysis (8) Breathlessness (5) Neck swelling (5)	Yes	T4N1M0	PTC	Subglottic mass occupying 80% of lumen	Jet ventilation	Yes	7.0	Yes	Injection laryngoplasty
2	58/M	Neck swelling (4) Breathlessness (4)	No	T4N1M0	PTC	Tracheal mass obstructing 50% of lumen	Oral intubation	No	6.0	Yes	-
3	67/F	Hemoptysis (24)	No	T4N0M0	PTC	Tracheal mass occupying 50% of lumen	Jet ventilation	Yes	6.5	No	-
4	74/M	Hemoptysis (2) Neck swelling (1)	No	T4N1M1	PTC	Tracheal mass occupying 50% of lumen	Jet ventilation	Yes	7.5	Yes	-
5	66/F	Neck swelling (2)	No	T4N1M0	PTC	Subglottic mass with minimal luminal obstruction	Oral intubation	No	6.5	Yes	-
6	44/F	Neck swelling (12) Breathlessness (3)	Yes	T4N0M0	FTC	Intraluminal subglottic lesion occupying 50% of lumen	Oral intubation	No	6.5	Yes	Non-selective reinnervation of recurrent laryngeal nerve with ansa hypoglossi

Surgery performed

**Figure 1:**
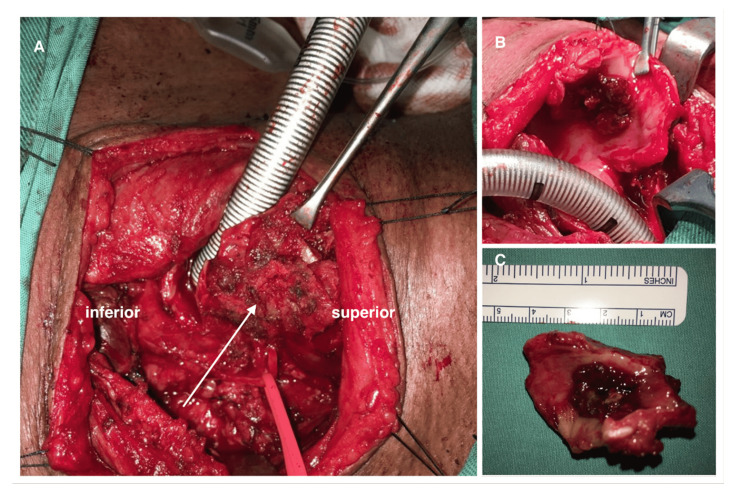
A. Tumor adherent to the anterior cricoid, first and second tracheal ring (arrow); B. tumor infiltration to subglottic region; C. excised tracheal tumor

**Figure 2:**
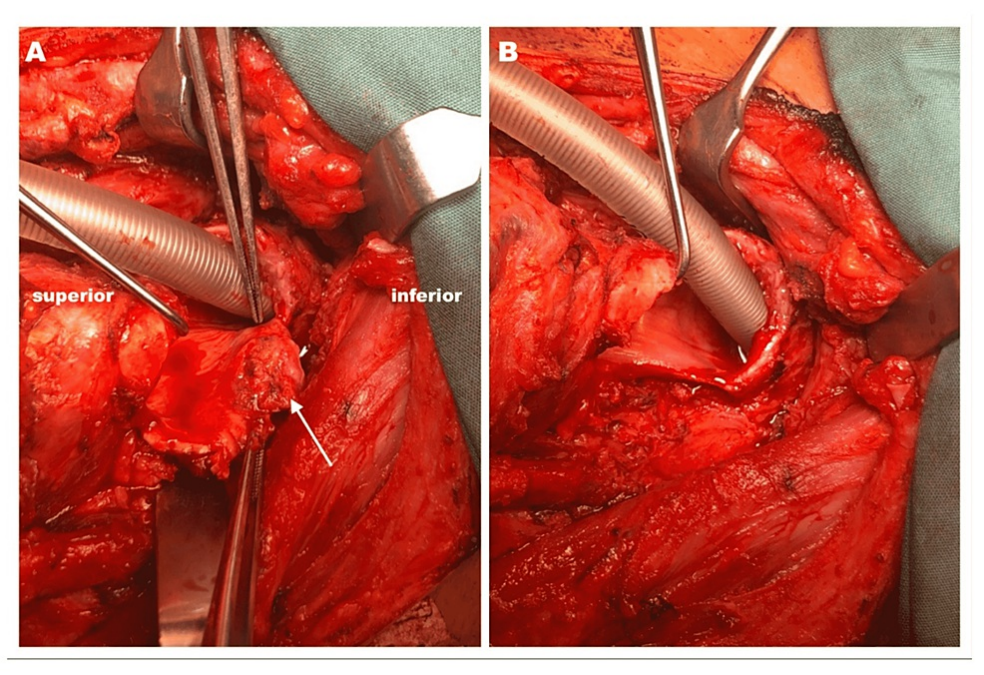
A. Intraluminal fungating lesion (arrow) at the first, second, third, and fourth anterolateral of right tracheal wall; B. post-tracheal sleeve resection

All patients underwent total thyroidectomy, cricotracheal sleeve resection with partial anastomosis, and tracheostomy. Intraluminal tumor debulking was done in three patients with significant airway narrowing to facilitate intubation. Primary anastomosis was done using Vicryl 3/0 and Prolene 3/0 sutures. Table 1 summarizes the operative procedures. Intraoperatively, all patients had tumor infiltration into the tracheal rings with Figures 1, 2 showing the intraoperative findings for two patients in this study. Table 2 summarizes the intraoperative findings.

**Table 2:** Intraoperative findings

Patient	Intraoperative findings
1	Fleshy subglottic mass arising from anterior subglottic region. Nodular left-sided thyroid mass measuring 3 x 4 cm infiltrating the cricoid cartilage, first tracheal ring and second tracheal ring (Figure 1). Left recurrent laryngeal nerve (RLN) encased by cervical lymph nodes.
2	Nodular left thyroid lobe tumor measuring 5 x 4 cm infiltrating the left side of first to third tracheal ring.
3	Nodular right thyroid lobe mass measuring 2 x 2 cm, infiltrating the second and third tracheal ring. Mass occupying two-thirds of the airway.
4	Right thyroid lobe mass infiltrating the first, second, third, and fourth tracheal ring of the right side (Figure 2).
5	Enlarged right thyroid gland, hard in consistency and infiltrating the strap muscles and right anterolateral wall of cricoid cartilage, first and second tracheal ring.
6	Left thyroid tumor measuring 8 x 10 cm, encasing the superior part of left RLN. Left RLN injured during dissection. Tumor adhered to posterolateral aspect of trachea and infiltrating tracheal lumen involving first and second tracheal ring.

Postoperative vocal fold mobility

Four patients had normal vocal fold mobility postoperatively. Two patients had iatrogenic vocal fold palsy. Patient 1 had an intraoperative injury to the left recurrent laryngeal nerve and was treated with injection laryngoplasty within the same setting. At one month postoperatively, this patient had left vocal fold palsy in paramedian position with mobile right vocal fold and was decannulated. Subsequent assessment at nine months postoperatively showed normal mobility of both vocal folds. Patient 5 had intraoperative left recurrent laryngeal nerve injury and underwent nonselective reinnervation with left ansa hypoglossi. The postoperative assessment demonstrated a left vocal fold in the median position with the mobile right vocal fold. Serial reassessment of the larynx up to 12 months demonstrated near-normal voice subjectively and objectively.

Decannulation

Prior to decannulation, all patients underwent flexible fiberoptic endoscopy under local anesthesia in the clinic to assess for the presence of an intraluminal tumor. After ensuring the absence of residual intraluminal tumor, a tracheostomy tube was occluded with a spigot and if patients could tolerate the spigot for two weeks, decannulation was done. Decannulation was successful in all patients postoperatively. Two patients were decannulated one month after surgery while the remaining four patients were decannulated six months after surgery.

Recurrence

All patients underwent radioactive iodine therapy postoperatively and have been free from recurrence till date.

Complications

One patient (16.6%) developed a neck hematoma requiring neck exploration and evacuation of the hematoma. Intraoperative injury to the recurrent laryngeal nerve was noted in two patients (33.3%) due to nerve encasement by the tumor. For these two patients, one was treated with nonselective reinnervation with left ansa hypoglossi while the other patient was managed with injection laryngoplasty. Both procedures were performed in the same setting as total thyroidectomy. One patient (16.6%) developed surgical emphysema, which was managed conservatively.

## Discussion

PTC comprises 89% of all thyroid carcinomas followed by FTC, which makes up 4.5% of cases [5]. Aerodigestive tract infiltration occurs in 1-16% of cases where the tumor invades the trachea, esophagus, or larynx once the thyroid gland capsule is breached [6]. This confers a poor prognosis with 45% 10-year survival rates in these patients in contrast to 91% in patients with no extracapsular tumor invasion [6]. Laryngotracheal involvement with intraluminal invasion through the full thickness of the tracheal mucosa, visible as a mass endoscopically, is categorized as stage IV in which the current guidelines recommend surgical resection when feasible [7,8]. All six patients in this series were categorized under stage IV.

The surgical techniques described in the literature include tracheal sleeve resection, circular wall resection with anastomosis, shave excision, laryngectomy, and tracheal resection with anastomosis [4]. Previously, laryngotracheal resection was controversial with a high risk of morbidity and mortality. Czaja and McCaffrey [4] demonstrated that there was no significant difference in survival rate between patients treated with laryngotracheal resection and those in whom the tumor was shaved off in cases with aerodigestive tract invasion. Tumor shaving from the upper aerodigestive tract allows maximal organ preservation while minimizing morbidity. However, this technique is unsuitable for tumors with deeper invasion due to the risk of residual gross disease or microscopic foci [9]. Thus, tumor shaving should be reserved for cases with superficial invasion not involving tracheal mucosa, namely Shin stage I and II [7-9].

A study by Tsai et al. [10] showed a five-year survival rate of 88% for patients who underwent laryngotracheal resection and 84% for those who underwent shave resection. Despite comparable survival rates, local recurrence was 8 times more likely in shave resection compared to laryngotracheal resection [10]. Thus, airway resection gives better tumor clearance but comes at the cost of higher airway complications such as edema, bleeding, infection, and anastomosis dehiscence [11]. Hence, following laryngotracheal resection, good postoperative monitoring and care preferably in an intensive care unit (ICU) is imperative. In our center, patients’ post-single-stage tracheal resection and anastomosis are intubated and ventilated in the ICU for a minimum of three days to provide stenting and prevent anastomosis dehiscence.

In this case series, the surgical technique performed was cricotracheal sleeve resection with partial closure and tracheostomy tube insertion. This surgery was chosen after taking into consideration the constraints of our center where ICU bed is limited. This technique was also best suited to the patients’ condition due to the urgency of performing the surgery as they were having airway compromise. Hence, delaying surgery for ICU bed availability was substandard. The patients were nursed in the normal ward postoperatively. All patients were followed up closely and there were no signs of intra-tracheal recurrence or severe laryngotracheal stenosis. All patients are well on follow-up and have been successfully decannulated.

The limitations of this study include a small sample size as not many patients with thyroid carcinoma have intraluminal laryngotracheal involvement. As this study was conducted in a single center, we believe a multicenter study will allow a larger patient group to corroborate our findings. We were not able to perform other types of resection techniques for comparison as it would require postoperative ICU monitoring which was lacking in our center.

## Conclusions

The prognosis of advanced thyroid carcinoma with aerodigestive tract invasion is good if complete surgical resection is feasible. Radical resection of the airway ensures improved tumor clearance but at the cost of significant morbidities to the patient which may be life-threatening. In this study, we were able to achieve good local disease control and low operative morbidity with a sleeve cricotracheal resection and insertion of tracheostomy (double-staged).

## References

[1] Olson E, Wintheiser G, Wolfe KM, Droessler J, Silberstein PT (2019). Epidemiology of thyroid cancer: a review of the National Cancer Database, 2000-2013. Cureus.

[2] Nixon IJ, Wang LY, Migliacci JC (2016). An international multi-institutional validation of age 55 years as a cutoff for risk stratification in the AJCC/UICC staging system for well-differentiated thyroid cancer. Thyroid.

[3] Andersen PE, Kinsella J, Loree TR, Shaha AR, Shah JP (1995). Differentiated carcinoma of the thyroid with extrathyroidal extension. Am J Surg.

[4] Czaja JM, McCaffrey TV (1997). The surgical management of laryngotracheal invasion by well-differentiated papillary thyroid carcinoma. Arch Otolaryngol Head Neck Surg.

[5] Haddad RI, Nasr C, Bischoff L (2018). NCCN guidelines insights: thyroid carcinoma, version 2.2018. J Natl Compr Canc Netw.

[6] Kim H, Jung HJ, Lee SY, Kwon TK, Kim KH, Sung MW, Hun Hah J (2016). Prognostic factors of locally invasive well-differentiated thyroid carcinoma involving the trachea. Eur Arch Otorhinolaryngol.

[7] Parida PK, Herkal K, Preetam C, Pradhan P, Samal DK, Sarkar S (2020). Analysis of pattern of laryngotracheal invasion by papillary thyroid carcinoma and their management: our experience. Indian J Otolaryngol Head Neck Surg.

[8] Shin DH, Mark EJ, Suen HC, Grillo HC (1993). Pathologic staging of papillary carcinoma of the thyroid with airway invasion based on the anatomic manner of extension to the trachea : a clinicopathologic study based on 22 patients who underwent thyroidectomy and airway resection. Hum Pathol.

[9] Hartl DM, Zago S, Leboulleux S, Mirghani H, Déandreis D, Baudin E, Schlumberger M (2014). Resection margins and prognosis in locally invasive thyroid cancer. Head Neck.

[10] Tsai YF, Tseng YL, Wu MH, Hung CJ, Lai WW, Lin MY (2005). Aggressive resection of the airway invaded by thyroid carcinoma. Br J Surg.

[11] Piazza C, Del Bon F, Barbieri D (2016). Tracheal and crico-tracheal resection and anastomosis for malignancies involving the thyroid gland and the airway. Ann Otol Rhinol Laryngol.

